# EXPERIENCES AND RECOMMENDATIONS FOR STARTING A UNIVERSITY GERONTOLOGY RESEARCH LABORATORY

**DOI:** 10.1093/geroni/igac059.403

**Published:** 2022-12-20

**Authors:** Britteny Howell, Jennifer Peterson

**Affiliations:** University of Alaska Anchorage, Anchorage, Alaska, United States; University of Alaska Fairbanks, Fairbanks, Alaska, United States

## Abstract

Although founding and directing an independent research laboratory is often expected of faculty at American universities, there are several barriers to successful completion of this important task. There is little guidance in the literature regarding exactly how to go about starting a research laboratory. The guidance that does exist for faculty often focuses on running research labs in the “hard sciences,” such as biomedical science and engineering, leaving social and behavioral scientists out of such considerations. Additionally, smaller or teaching-focused universities often have little infrastructure or support for starting a research lab, so faculty at these institutions may not know where to begin. These barriers are significant concerns for junior faculty, who are often unprepared for the realities and challenges of starting a successful research lab while obtaining other milestones required of promotion and tenure. We present two examples of recently-formed gerontology research laboratories begun by junior faculty, one in the psychology department of a research university and one in public health at a teaching-focused university. Our case studies present the audience with specific examples, lessons learned, and guidance for starting their own gerontology research laboratory in higher education, as well as recommendations on maintaining its functioning during a global pandemic (COVID-19).

